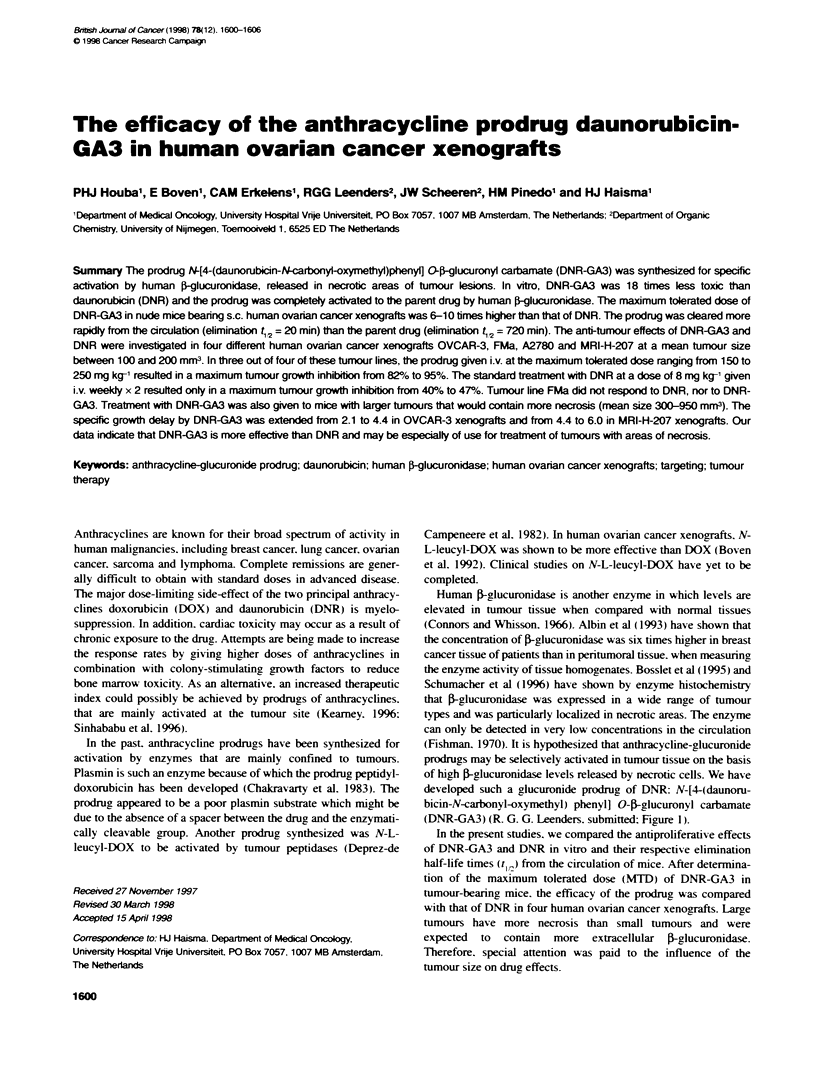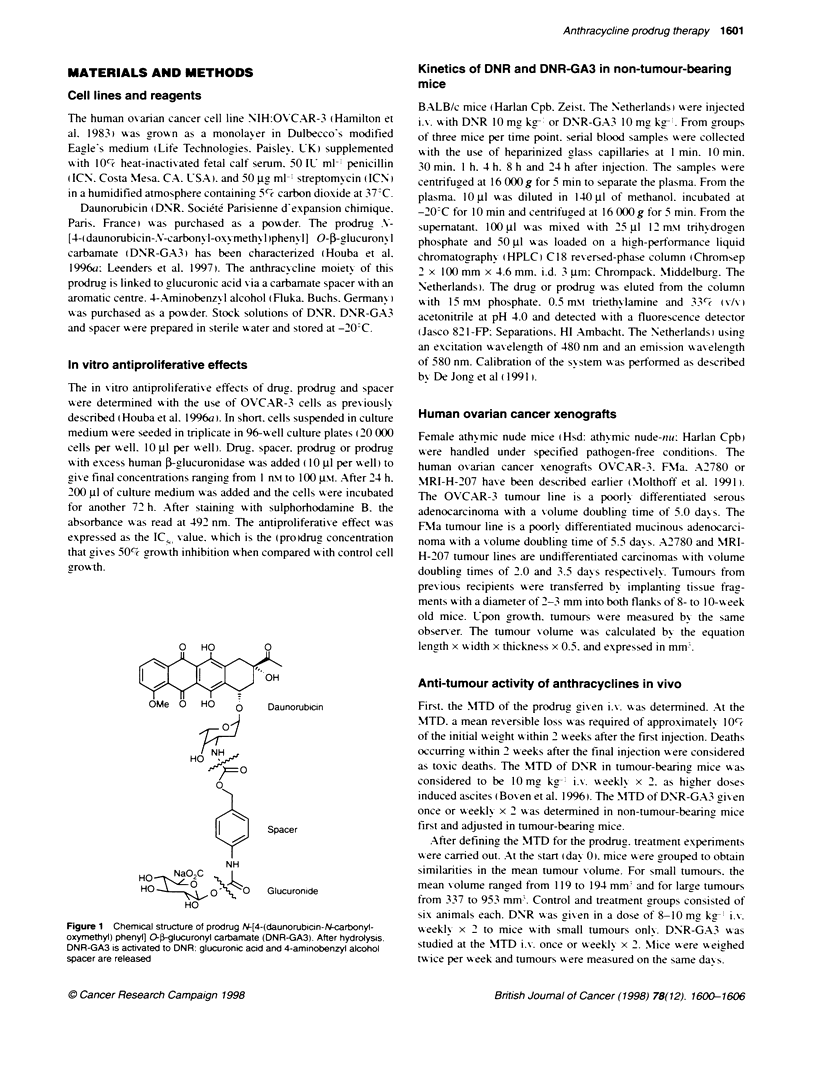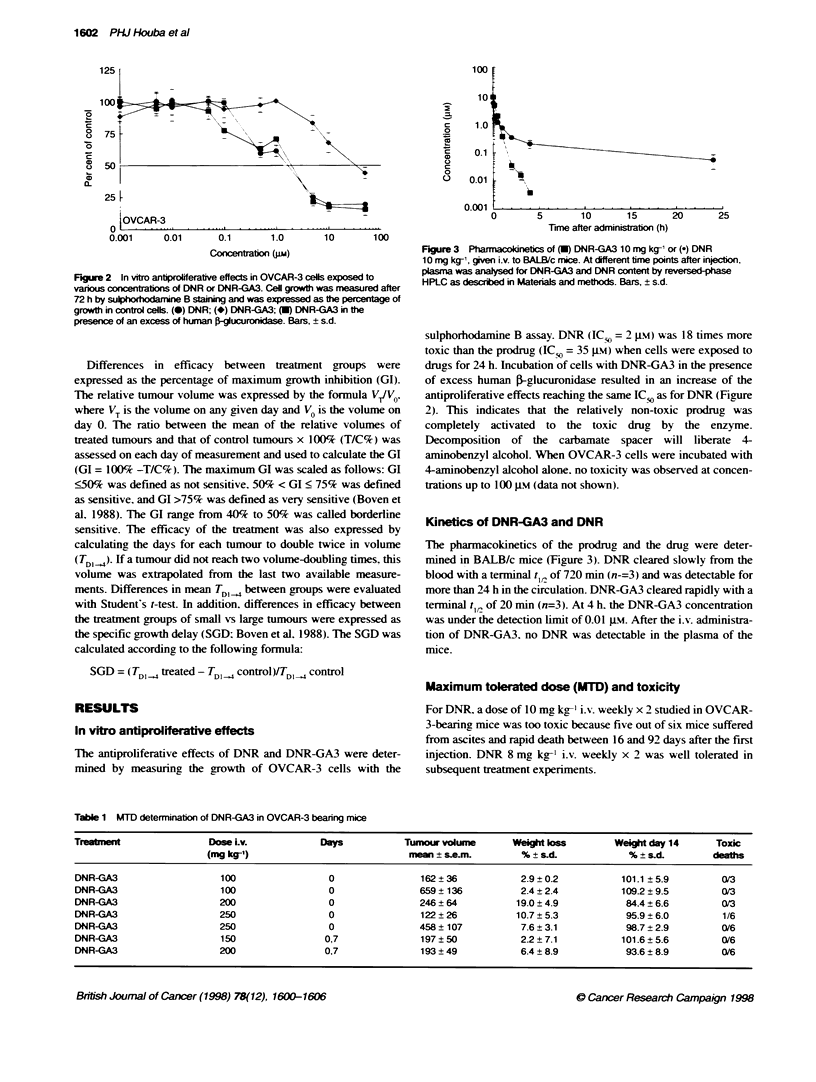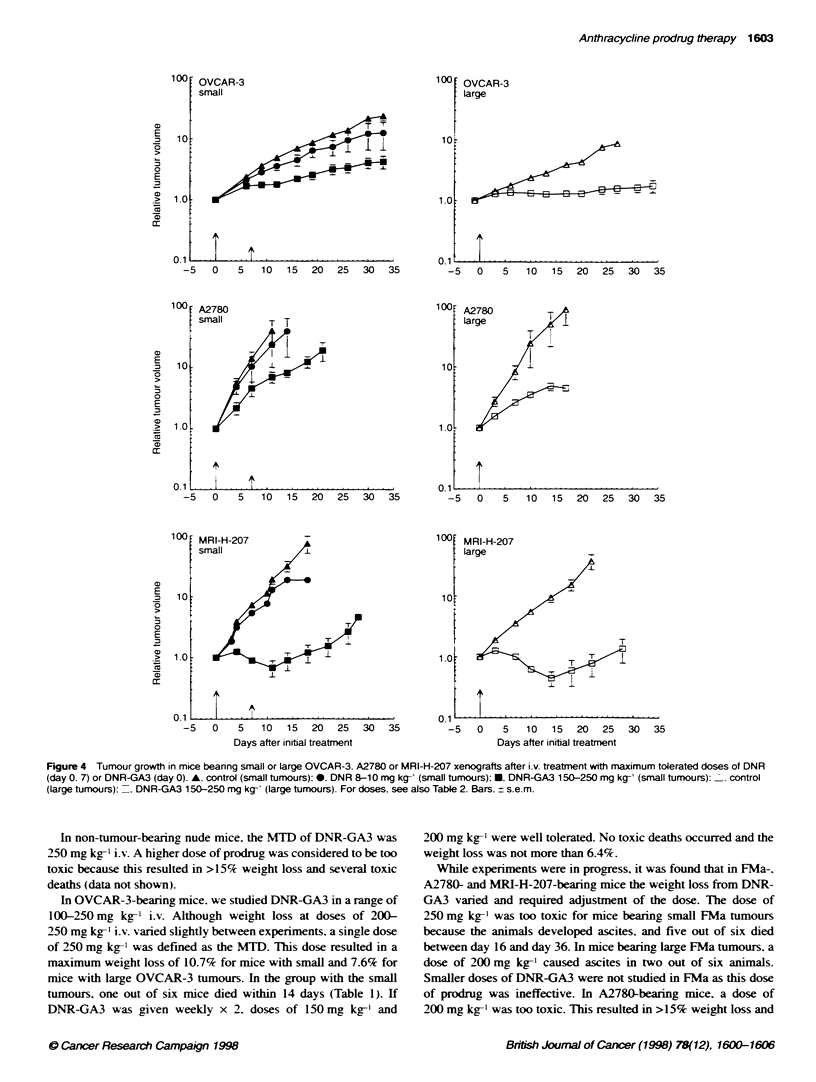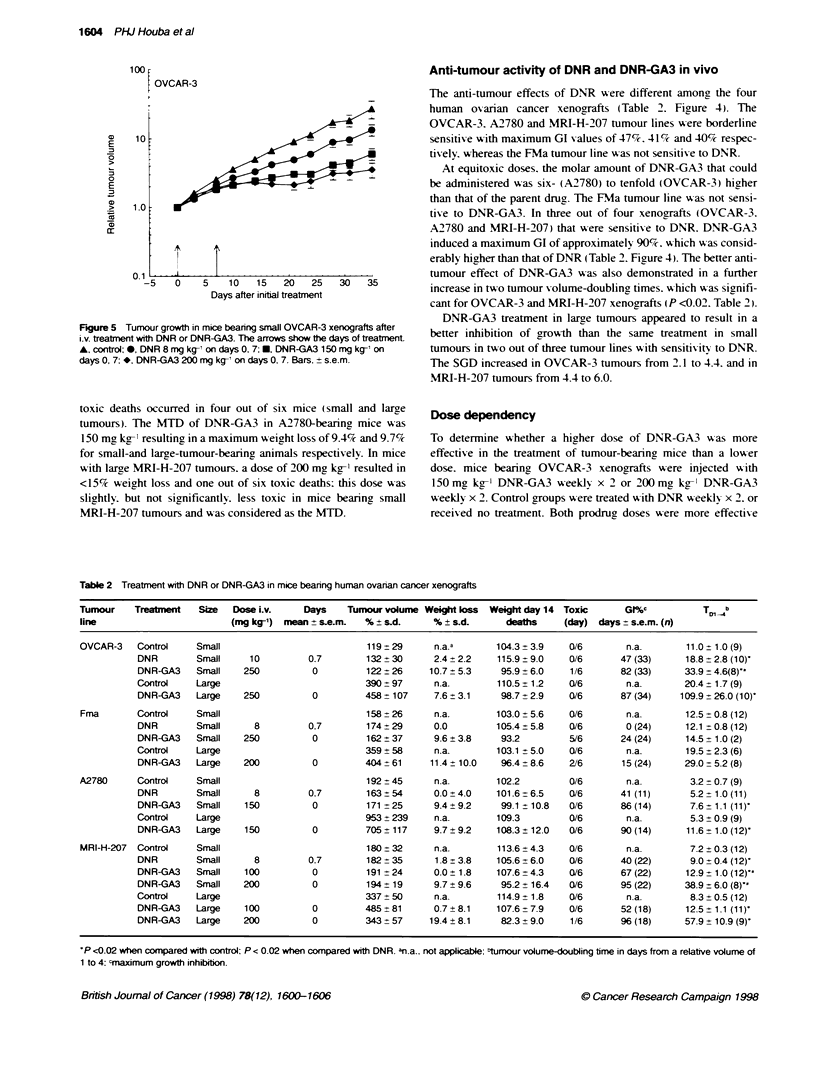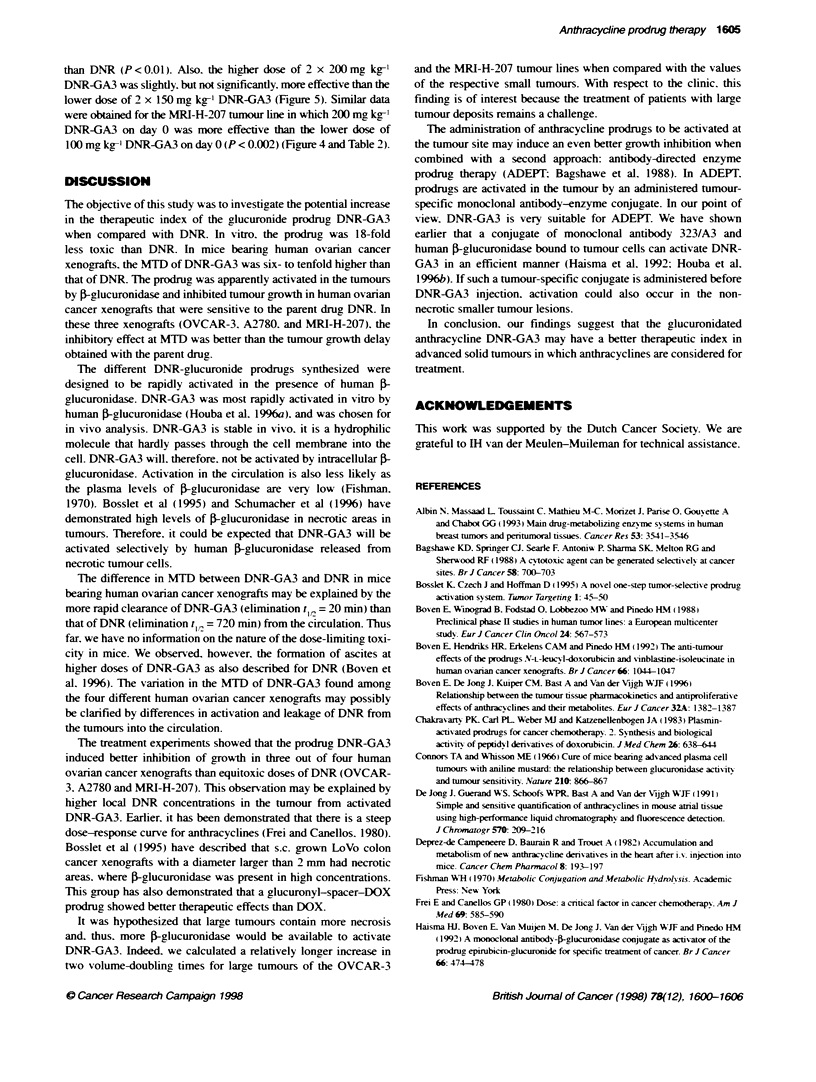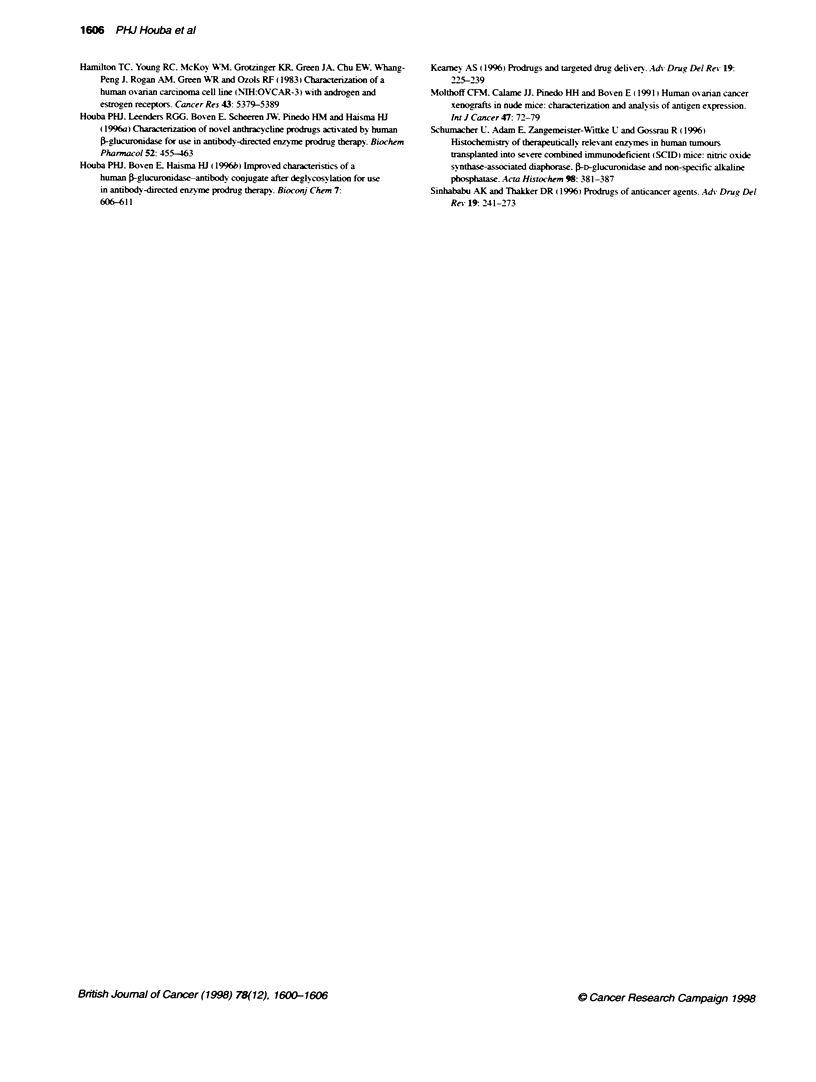# The efficacy of the anthracycline prodrug daunorubicin-GA3 in human ovarian cancer xenografts.

**DOI:** 10.1038/bjc.1998.729

**Published:** 1998-12

**Authors:** P. H. Houba, E. Boven, C. A. Erkelens, R. G. Leenders, J. W. Scheeren, H. M. Pinedo, H. J. Haisma

**Affiliations:** Department of Medical Oncology, University Hospital Vrije Universiteit, Amsterdam, The Netherlands.

## Abstract

The prodrug N-[4-(daunorubicin-N-carbonyl-oxymethyl)phenyl] O-beta-glucuronyl carbamate (DNR-GA3) was synthesized for specific activation by human beta-glucuronidase, released in necrotic areas of tumour lesions. In vitro, DNR-GA3 was 18 times less toxic than daunorubicin (DNR) and the prodrug was completely activated to the parent drug by human beta-glucuronidase. The maximum tolerated dose of DNR-GA3 in nude mice bearing s.c. human ovarian cancer xenografts was 6-10 times higher than that of DNR. The prodrug was cleared more rapidly from the circulation (elimination t1/2 = 20 min) than the parent drug (elimination t1/2 = 720 min). The anti-tumour effects of DNR-GA3 and DNR were investigated in four different human ovarian cancer xenografts OVCAR-3, FMa, A2780 and MRI-H-207 at a mean tumour size between 100 and 200 mm3. In three out of four of these tumour lines, the prodrug given i.v. at the maximum tolerated dose ranging from 150 to 250 mg kg(-1) resulted in a maximum tumour growth inhibition from 82% to 95%. The standard treatment with DNR at a dose of 8 mg kg(-1) given i.v. weekly x 2 resulted only in a maximum tumour growth inhibition from 40% to 47%. Tumour line FMa did not respond to DNR, nor to DNR-GA3. Treatment with DNR-GA3 was also given to mice with larger tumours that would contain more necrosis (mean size 300-950 mm3). The specific growth delay by DNR-GA3 was extended from 2.1 to 4.4 in OVCAR-3 xenografts and from 4.4 to 6.0 in MRI-H-207 xenografts. Our data indicate that DNR-GA3 is more effective than DNR and may be especially of use for treatment of tumours with areas of necrosis.


British Journal of Cancer (1998) 78(12), 1600–1606
© 1998 Cancer Research Campaign

### The efficacy of the anthracycline prodrug daunorubicin-GA3 in human ovarian cancer xenografts

PHJ Houba[1], E Boven[1], CAM Erkelens[1], RGG Leenders[2], JW Scheeren[2], HM Pinedo[1] and HJ Haisma[1]

[1]Department of Medical Oncology, University Hospital Vrije Universiteit, PO Box 7057, 1007 MB Amsterdam, The Netherlands; [2]Department of Organic Chemistry, University of Nijmegen, Toernooiveld 1, 6525 ED The Netherlands

**Summary** The prodrug *N*-[4-(daunorubicin-*N*-carbonyl-oxymethyl)phenyl] *O*-β-glucuronyl carbamate (DNR-GA3) was synthesized for specific activation by human β-glucuronidase, released in necrotic areas of tumour lesions. In vitro, DNR-GA3 was 18 times less toxic than daunorubicin (DNR) and the prodrug was completely activated to the parent drug by human β-glucuronidase. The maximum tolerated dose of DNR-GA3 in nude mice bearing s.c. human ovarian cancer xenografts was 6–10 times higher than that of DNR. The prodrug was cleared more rapidly from the circulation (elimination $t_{1/2}$ = 20 min) than the parent drug (elimination $t_{1/2}$ = 720 min). The anti-tumour effects of DNR-GA3 and DNR were investigated in four different human ovarian cancer xenografts OVCAR-3, FMa, A2780 and MRI-H-207 at a mean tumour size between 100 and 200 mm$^3$. In three out of four of these tumour lines, the prodrug given i.v. at the maximum tolerated dose ranging from 150 to 250 mg kg$^{-1}$ resulted in a maximum tumour growth inhibition from 82% to 95%. The standard treatment with DNR at a dose of 8 mg kg$^{-1}$ given i.v. weekly × 2 resulted only in a maximum tumour growth inhibition from 40% to 47%. Tumour line FMa did not respond to DNR, nor to DNR-GA3. Treatment with DNR-GA3 was also given to mice with larger tumours that would contain more necrosis (mean size 300–950 mm$^3$). The specific growth delay by DNR-GA3 was extended from 2.1 to 4.4 in OVCAR-3 xenografts and from 4.4 to 6.0 in MRI-H-207 xenografts. Our data indicate that DNR-GA3 is more effective than DNR and may be especially of use for treatment of tumours with areas of necrosis.

**Keywords:** anthracycline-glucuronide prodrug; daunorubicin; human β-glucuronidase; human ovarian cancer xenografts; targeting; tumour therapy

Anthracyclines are known for their broad spectrum of activity in human malignancies, including breast cancer, lung cancer, ovarian cancer, sarcoma and lymphoma. Complete remissions are generally difficult to obtain with standard doses in advanced disease. The major dose-limiting side-effect of the two principal anthracyclines doxorubicin (DOX) and daunorubicin (DNR) is myelosuppression. In addition, cardiac toxicity may occur as a result of chronic exposure to the drug. Attempts are being made to increase the response rates by giving higher doses of anthracyclines in combination with colony-stimulating growth factors to reduce bone marrow toxicity. As an alternative, an increased therapeutic index could possibly be achieved by prodrugs of anthracyclines, that are mainly activated at the tumour site (Kearney, 1996; Sinhababu et al, 1996).

In the past, anthracycline prodrugs have been synthesized for activation by enzymes that are mainly confined to tumours. Plasmin is such an enzyme because of which the prodrug peptidyl-doxorubicin has been developed (Chakravarty et al, 1983). The prodrug appeared to be a poor plasmin substrate which might be due to the absence of a spacer between the drug and the enzymatically cleavable group. Another prodrug synthesized was *N*-L-leucyl-DOX to be activated by tumour peptidases (Deprez-de Campeneere et al, 1982). In human ovarian cancer xenografts, *N*-L-leucyl-DOX was shown to be more effective than DOX (Boven et al, 1992). Clinical studies on *N*-L-leucyl-DOX have yet to be completed.

Human β-glucuronidase is another enzyme in which levels are elevated in tumour tissue when compared with normal tissues (Connors and Whisson, 1966). Albin et al (1993) have shown that the concentration of β-glucuronidase was six times higher in breast cancer tissue of patients than in peritumoral tissue, when measuring the enzyme activity of tissue homogenates. Bosslet et al (1995) and Schumacher et al (1996) have shown by enzyme histochemistry that β-glucuronidase was expressed in a wide range of tumour types and was particularly localized in necrotic areas. The enzyme can only be detected in very low concentrations in the circulation (Fishman, 1970). It is hypothesized that anthracycline-glucuronide prodrugs may be selectively activated in tumour tissue on the basis of high β-glucuronidase levels released by necrotic cells. We have developed such a glucuronide prodrug of DNR: *N*-[4-(daunorubicin-*N*-carbonyl-oxymethyl) phenyl] *O*-β-glucuronyl carbamate (DNR-GA3) (R. G. G. Leenders, submitted; Figure 1).

In the present studies, we compared the antiproliferative effects of DNR-GA3 and DNR in vitro and their respective elimination half-life times ($t_{1/2}$) from the circulation of mice. After determination of the maximum tolerated dose (MTD) of DNR-GA3 in tumour-bearing mice, the efficacy of the prodrug was compared with that of DNR in four human ovarian cancer xenografts. Large tumours have more necrosis than small tumours and were expected to contain more extracellular β-glucuronidase. Therefore, special attention was paid to the influence of the tumour size on drug effects.

*Received 27 November 1997*
*Revised 30 March 1998*
*Accepted 15 April 1998*

*Correspondence to:* HJ Haisma, Department of Medical Oncology, University Hospital Vrije Universiteit, PO Box 7057, 1007 MB Amsterdam, The Netherlands

### MATERIALS AND METHODS

### Cell lines and reagents

The human ovarian cancer cell line NIH:OVCAR-3 (Hamilton et al. 1983) was grown as a monolayer in Dulbecco's modified Eagle's medium (Life Technologies, Paisley, UK) supplemented with 10% heat-inactivated fetal calf serum, 50 IU ml$^{-1}$ penicillin (ICN, Costa Mesa, CA, USA), and 50 µg ml$^{-1}$ streptomycin (ICN) in a humidified atmosphere containing 5% carbon dioxide at 37°C.

Daunorubicin (DNR, Société Parisienne d'expansion chimique, Paris, France) was purchased as a powder. The prodrug *N*-[4-(daunorubicin-*N*-carbonyl-oxymethyl)phenyl] *O*-β-glucuronyl carbamate (DNR-GA3) has been characterized (Houba et al. 1996*a*; Leenders et al. 1997). The anthracycline moiety of this prodrug is linked to glucuronic acid via a carbamate spacer with an aromatic centre. 4-Aminobenzyl alcohol (Fluka, Buchs, Germany) was purchased as a powder. Stock solutions of DNR, DNR-GA3 and spacer were prepared in sterile water and stored at –20°C.

### In vitro antiproliferative effects

The in vitro antiproliferative effects of drug, prodrug and spacer were determined with the use of OVCAR-3 cells as previously described (Houba et al. 1996*a*). In short, cells suspended in culture medium were seeded in triplicate in 96-well culture plates (20 000 cells per well, 10 µl per well). Drug, spacer, prodrug or prodrug with excess human β-glucuronidase was added (10 µl per well) to give final concentrations ranging from 1 nM to 100 µM. After 24 h, 200 µl of culture medium was added and the cells were incubated for another 72 h. After staining with sulphorhodamine B, the absorbance was read at 492 nm. The antiproliferative effect was expressed as the $IC_{50}$ value, which is the (pro)drug concentration that gives 50% growth inhibition when compared with control cell growth.

**Figure 1** Chemical structure of prodrug *N*-[4-(daunorubicin-*N*-carbonyl-oxymethyl) phenyl] *O*-β-glucuronyl carbamate (DNR-GA3). After hydrolysis, DNR-GA3 is activated to DNR; glucuronic acid and 4-aminobenzyl alcohol spacer are released

### Kinetics of DNR and DNR-GA3 in non-tumour-bearing mice

BALB/c mice (Harlan Cpb, Zeist, The Netherlands) were injected i.v. with DNR 10 mg kg$^{-1}$ or DNR-GA3 10 mg kg$^{-1}$. From groups of three mice per time point, serial blood samples were collected with the use of heparinized glass capillaries at 1 min, 10 min, 30 min, 1 h, 4 h, 8 h and 24 h after injection. The samples were centrifuged at 16 000 *g* for 5 min to separate the plasma. From the plasma, 10 µl was diluted in 140 µl of methanol, incubated at –20°C for 10 min and centrifuged at 16 000 *g* for 5 min. From the supernatant, 100 µl was mixed with 25 µl 12 mM trihydrogen phosphate and 50 µl was loaded on a high-performance liquid chromatography (HPLC) C18 reversed-phase column (Chromsep 2 × 100 mm × 4.6 mm, i.d. 3 µm; Chrompack, Middelburg, The Netherlands). The drug or prodrug was eluted from the column with 15 mM phosphate, 0.5 mM triethylamine and 33% (v/v) acetonitrile at pH 4.0 and detected with a fluorescence detector (Jasco 821-FP; Separations, HI Ambacht, The Netherlands) using an excitation wavelength of 480 nm and an emission wavelength of 580 nm. Calibration of the system was performed as described by De Jong et al (1991).

### Human ovarian cancer xenografts

Female athymic nude mice (Hsd: athymic nude-*nu*; Harlan Cpb) were handled under specified pathogen-free conditions. The human ovarian cancer xenografts OVCAR-3, FMa, A2780 or MRI-H-207 have been described earlier (Molthoff et al. 1991). The OVCAR-3 tumour line is a poorly differentiated serous adenocarcinoma with a volume doubling time of 5.0 days. The FMa tumour line is a poorly differentiated mucinous adenocarcinoma with a volume doubling time of 5.5 days. A2780 and MRI-H-207 tumour lines are undifferentiated carcinomas with volume doubling times of 2.0 and 3.5 days respectively. Tumours from previous recipients were transferred by implanting tissue fragments with a diameter of 2–3 mm into both flanks of 8- to 10-week old mice. Upon growth, tumours were measured by the same observer. The tumour volume was calculated by the equation length × width × thickness × 0.5, and expressed in mm$^3$.

### Anti-tumour activity of anthracyclines in vivo

First, the MTD of the prodrug given i.v. was determined. At the MTD, a mean reversible loss was required of approximately 10% of the initial weight within 2 weeks after the first injection. Deaths occurring within 2 weeks after the final injection were considered as toxic deaths. The MTD of DNR in tumour-bearing mice was considered to be 10 mg kg$^{-1}$ i.v. weekly × 2, as higher doses induced ascites (Boven et al. 1996). The MTD of DNR-GA3 given once or weekly × 2 was determined in non-tumour-bearing mice first and adjusted in tumour-bearing mice.

After defining the MTD for the prodrug, treatment experiments were carried out. At the start (day 0), mice were grouped to obtain similarities in the mean tumour volume. For small tumours, the mean volume ranged from 119 to 194 mm$^3$ and for large tumours from 337 to 953 mm$^3$. Control and treatment groups consisted of six animals each. DNR was given in a dose of 8–10 mg kg$^{-1}$ i.v. weekly × 2 to mice with small tumours only. DNR-GA3 was studied at the MTD i.v. once or weekly × 2. Mice were weighed twice per week and tumours were measured on the same days.

© Cancer Research Campaign 1998

**Figure 2** In vitro antiproliferative effects in OVCAR-3 cells exposed to various concentrations of DNR or DNR-GA3. Cell growth was measured after 72 h by sulphorhodamine B staining and was expressed as the percentage of growth in control cells. (●) DNR; (◆) DNR-GA3; (■) DNR-GA3 in the presence of an excess of human β-glucuronidase. Bars, ± s.d.

**Figure 3** Pharmacokinetics of (■) DNR-GA3 10 mg kg$^{-1}$ or (•) DNR 10 mg kg$^{-1}$, given i.v. to BALB/c mice. At different time points after injection, plasma was analysed for DNR-GA3 and DNR content by reversed-phase HPLC as described in Materials and methods. Bars, ± s.d.

Differences in efficacy between treatment groups were expressed as the percentage of maximum growth inhibition (GI). The relative tumour volume was expressed by the formula $V_T/V_0$, where $V_T$ is the volume on any given day and $V_0$ is the volume on day 0. The ratio between the mean of the relative volumes of treated tumours and that of control tumours × 100% (T/C%) was assessed on each day of measurement and used to calculate the GI (GI = 100% –T/C%). The maximum GI was scaled as follows: GI ≤50% was defined as not sensitive, 50% < GI ≤ 75% was defined as sensitive, and GI >75% was defined as very sensitive (Boven et al, 1988). The GI range from 40% to 50% was called borderline sensitive. The efficacy of the treatment was also expressed by calculating the days for each tumour to double twice in volume ($T_{D1\rightarrow4}$). If a tumour did not reach two volume-doubling times, this volume was extrapolated from the last two available measurements. Differences in mean $T_{D1\rightarrow4}$ between groups were evaluated with Student's *t*-test. In addition, differences in efficacy between the treatment groups of small vs large tumours were expressed as the specific growth delay (SGD; Boven et al, 1988). The SGD was calculated according to the following formula:

$$SGD = (T_{D1\rightarrow4}\text{ treated} - T_{D1\rightarrow4}\text{ control})/T_{D1\rightarrow4}\text{ control}$$

### RESULTS

### In vitro antiproliferative effects

The antiproliferative effects of DNR and DNR-GA3 were determined by measuring the growth of OVCAR-3 cells with the sulphorhodamine B assay. DNR ($IC_{50}$ = 2 μM) was 18 times more toxic than the prodrug ($IC_{50}$ = 35 μM) when cells were exposed to drugs for 24 h. Incubation of cells with DNR-GA3 in the presence of excess human β-glucuronidase resulted in an increase of the antiproliferative effects reaching the same $IC_{50}$ as for DNR (Figure 2). This indicates that the relatively non-toxic prodrug was completely activated to the toxic drug by the enzyme. Decomposition of the carbamate spacer will liberate 4-aminobenzyl alcohol. When OVCAR-3 cells were incubated with 4-aminobenzyl alcohol alone, no toxicity was observed at concentrations up to 100 μM (data not shown).

### Kinetics of DNR-GA3 and DNR

The pharmacokinetics of the prodrug and the drug were determined in BALB/c mice (Figure 3). DNR cleared slowly from the blood with a terminal $t_{1/2}$ of 720 min (*n*-=3) and was detectable for more than 24 h in the circulation. DNR-GA3 cleared rapidly with a terminal $t_{1/2}$ of 20 min (*n*=3). At 4 h, the DNR-GA3 concentration was under the detection limit of 0.01 μM. After the i.v. administration of DNR-GA3, no DNR was detectable in the plasma of the mice.

### Maximum tolerated dose (MTD) and toxicity

For DNR, a dose of 10 mg kg$^{-1}$ i.v. weekly × 2 studied in OVCAR-3-bearing mice was too toxic because five out of six mice suffered from ascites and rapid death between 16 and 92 days after the first injection. DNR 8 mg kg$^{-1}$ i.v. weekly × 2 was well tolerated in subsequent treatment experiments.

**Table 1** MTD determination of DNR-GA3 in OVCAR-3 bearing mice

| Treatment | Dose i.v. (mg kg$^{-1}$) | Days | Tumour volume mean ± s.e.m. | Weight loss % ± s.d. | Weight day 14 % ± s.d. | Toxic deaths |
|---|---|---|---|---|---|---|
| DNR-GA3 | 100 | 0 | 162 ± 36 | 2.9 ± 0.2 | 101.1 ± 5.9 | 0/3 |
| DNR-GA3 | 100 | 0 | 659 ± 136 | 2.4 ± 2.4 | 109.2 ± 9.5 | 0/3 |
| DNR-GA3 | 200 | 0 | 246 ± 64 | 19.0 ± 4.9 | 84.4 ± 6.6 | 0/3 |
| DNR-GA3 | 250 | 0 | 122 ± 26 | 10.7 ± 5.3 | 95.9 ± 6.0 | 1/6 |
| DNR-GA3 | 250 | 0 | 458 ± 107 | 7.6 ± 3.1 | 98.7 ± 2.9 | 0/6 |
| DNR-GA3 | 150 | 0,7 | 197 ± 50 | 2.2 ± 7.1 | 101.6 ± 5.6 | 0/6 |
| DNR-GA3 | 200 | 0,7 | 193 ± 49 | 6.4 ± 8.9 | 93.6 ± 8.9 | 0/6 |

© Cancer Research Campaign 1998

**Figure 4** Tumour growth in mice bearing small or large OVCAR-3, A2780 or MRI-H-207 xenografts after i.v. treatment with maximum tolerated doses of DNR (day 0, 7) or DNR-GA3 (day 0). ▲, control (small tumours); ●, DNR 8–10 mg $kg^{-1}$ (small tumours); ■, DNR-GA3 150–250 mg $kg^{-1}$ (small tumours); △, control (large tumours); □, DNR-GA3 150–250 mg $kg^{-1}$ (large tumours). For doses, see also Table 2. Bars, ± s.e.m.

In non-tumour-bearing nude mice, the MTD of DNR-GA3 was 250 mg $kg^{-1}$ i.v. A higher dose of prodrug was considered to be too toxic because this resulted in >15% weight loss and several toxic deaths (data not shown).

In OVCAR-3-bearing mice, we studied DNR-GA3 in a range of 100–250 mg $kg^{-1}$ i.v. Although weight loss at doses of 200–250 mg $kg^{-1}$ i.v. varied slightly between experiments, a single dose of 250 mg $kg^{-1}$ was defined as the MTD. This dose resulted in a maximum weight loss of 10.7% for mice with small and 7.6% for mice with large OVCAR-3 tumours. In the group with the small tumours, one out of six mice died within 14 days (Table 1). If DNR-GA3 was given weekly × 2, doses of 150 mg $kg^{-1}$ and 200 mg $kg^{-1}$ were well tolerated. No toxic deaths occurred and the weight loss was not more than 6.4%.

While experiments were in progress, it was found that in FMa-, A2780- and MRI-H-207-bearing mice the weight loss from DNR-GA3 varied and required adjustment of the dose. The dose of 250 mg $kg^{-1}$ was too toxic for mice bearing small FMa tumours because the animals developed ascites, and five out of six died between day 16 and day 36. In mice bearing large FMa tumours, a dose of 200 mg $kg^{-1}$ caused ascites in two out of six animals. Smaller doses of DNR-GA3 were not studied in FMa as this dose of prodrug was ineffective. In A2780-bearing mice, a dose of 200 mg $kg^{-1}$ was too toxic. This resulted in >15% weight loss and

**Figure 5** Tumour growth in mice bearing small OVCAR-3 xenografts after i.v. treatment with DNR or DNR-GA3. The arrows show the days of treatment. ▲, control; ●, DNR 8 mg kg$^{-1}$ on days 0, 7; ■, DNR-GA3 150 mg kg$^{-1}$ on days 0, 7; ◆, DNR-GA3 200 mg kg$^{-1}$ on days 0, 7. Bars, ± s.e.m.

toxic deaths occurred in four out of six mice (small and large tumours). The MTD of DNR-GA3 in A2780-bearing mice was 150 mg kg$^{-1}$ resulting in a maximum weight loss of 9.4% and 9.7% for small-and large-tumour-bearing animals respectively. In mice with large MRI-H-207 tumours, a dose of 200 mg kg$^{-1}$ resulted in <15% weight loss and one out of six toxic deaths; this dose was slightly, but not significantly, less toxic in mice bearing small MRI-H-207 tumours and was considered as the MTD.

### Anti-tumour activity of DNR and DNR-GA3 in vivo

The anti-tumour effects of DNR were different among the four human ovarian cancer xenografts (Table 2, Figure 4). The OVCAR-3, A2780 and MRI-H-207 tumour lines were borderline sensitive with maximum GI values of 47%, 41% and 40% respectively, whereas the FMa tumour line was not sensitive to DNR.

At equitoxic doses, the molar amount of DNR-GA3 that could be administered was six- (A2780) to tenfold (OVCAR-3) higher than that of the parent drug. The FMa tumour line was not sensitive to DNR-GA3. In three out of four xenografts (OVCAR-3, A2780 and MRI-H-207) that were sensitive to DNR, DNR-GA3 induced a maximum GI of approximately 90%, which was considerably higher than that of DNR (Table 2, Figure 4). The better anti-tumour effect of DNR-GA3 was also demonstrated in a further increase in two tumour volume-doubling times, which was significant for OVCAR-3 and MRI-H-207 xenografts ($P$ <0.02, Table 2).

DNR-GA3 treatment in large tumours appeared to result in a better inhibition of growth than the same treatment in small tumours in two out of three tumour lines with sensitivity to DNR. The SGD increased in OVCAR-3 tumours from 2.1 to 4.4, and in MRI-H-207 tumours from 4.4 to 6.0.

### Dose dependency

To determine whether a higher dose of DNR-GA3 was more effective in the treatment of tumour-bearing mice than a lower dose, mice bearing OVCAR-3 xenografts were injected with 150 mg kg$^{-1}$ DNR-GA3 weekly × 2 or 200 mg kg$^{-1}$ DNR-GA3 weekly × 2. Control groups were treated with DNR weekly × 2, or received no treatment. Both prodrug doses were more effective

**Table 2** Treatment with DNR or DNR-GA3 in mice bearing human ovarian cancer xenografts

| Tumour line | Treatment | Size | Dose i.v. (mg kg$^{-1}$) | Days mean ± s.e.m. | Tumour volume % ± s.d. | Weight loss % ± s.d. | Weight day 14 deaths | Toxic (day) | GI%[c] days ± s.e.m. ($n$) | $T_{D1-4}$[b] |
|---|---|---|---|---|---|---|---|---|---|---|
| OVCAR-3 | Control | Small | | | 119 ± 29 | n.a.[a] | 104.3 ± 3.9 | 0/6 | n.a. | 11.0 ± 1.0 (9) |
| | DNR | Small | 10 | 0.7 | 132 ± 30 | 2.4 ± 2.2 | 115.9 ± 9.0 | 0/6 | 47 (33) | 18.8 ± 2.8 (10)* |
| | DNR-GA3 | Small | 250 | 0 | 122 ± 26 | 10.7 ± 5.3 | 95.9 ± 6.0 | 1/6 | 82 (33) | 33.9 ± 4.6(8)** |
| | Control | Large | | | 390 ± 97 | n.a. | 110.5 ± 1.2 | 0/6 | n.a. | 20.4 ± 1.7 (9) |
| | DNR-GA3 | Large | 250 | 0 | 458 ± 107 | 7.6 ± 3.1 | 98.7 ± 2.9 | 0/6 | 87 (34) | 109.9 ± 26.0 (10)* |
| Fma | Control | Small | | | 158 ± 26 | n.a. | 103.0 ± 5.6 | 0/6 | n.a. | 12.5 ± 0.8 (12) |
| | DNR | Small | 8 | 0.7 | 174 ± 29 | 0.0 | 105.4 ± 5.8 | 0/6 | 0 (24) | 12.1 ± 0.8 (12) |
| | DNR-GA3 | Small | 250 | 0 | 162 ± 37 | 9.6 ± 3.8 | 93.2 | 5/6 | 24 (24) | 14.5 ± 1.0 (2) |
| | Control | Large | | | 359 ± 58 | n.a. | 103.1 ± 5.0 | 0/6 | n.a. | 19.5 ± 2.3 (6) |
| | DNR-GA3 | Large | 200 | 0 | 404 ± 61 | 11.4 ± 10.0 | 96.4 ± 8.6 | 2/6 | 15 (24) | 29.0 ± 5.2 (8) |
| A2780 | Control | Small | | | 192 ± 45 | n.a. | 102.2 | 0/6 | n.a. | 3.2 ± 0.7 (9) |
| | DNR | Small | 8 | 0.7 | 163 ± 54 | 0.0 ± 4.0 | 101.6 ± 6.5 | 0/6 | 41 (11) | 5.2 ± 1.0 (11) |
| | DNR-GA3 | Small | 150 | 0 | 171 ± 25 | 9.4 ± 9.2 | 99.1 ± 10.8 | 0/6 | 86 (14) | 7.6 ± 1.1 (11)* |
| | Control | Large | | | 953 ± 239 | n.a. | 109.3 | 0/6 | n.a. | 5.3 ± 0.9 (9) |
| | DNR-GA3 | Large | 150 | 0 | 705 ± 117 | 9.7 ± 9.2 | 108.3 ± 12.0 | 0/6 | 90 (14) | 11.6 ± 1.0 (12)* |
| MRI-H-207 | Control | Small | | | 180 ± 32 | n.a. | 113.6 ± 4.3 | 0/6 | n.a. | 7.2 ± 0.3 (12) |
| | DNR | Small | 8 | 0.7 | 182 ± 35 | 1.8 ± 3.8 | 105.6 ± 6.0 | 0/6 | 40 (22) | 9.0 ± 0.4 (12)* |
| | DNR-GA3 | Small | 100 | 0 | 191 ± 24 | 0.0 ± 1.8 | 107.6 ± 4.3 | 0/6 | 67 (22) | 12.9 ± 1.0 (12)** |
| | DNR-GA3 | Small | 200 | 0 | 194 ± 19 | 9.7 ± 9.6 | 95.2 ± 16.4 | 0/6 | 95 (22) | 38.9 ± 6.0 (8)** |
| | Control | Large | | | 337 ± 50 | n.a. | 114.9 ± 1.8 | 0/6 | n.a. | 8.3 ± 0.5 (12) |
| | DNR-GA3 | Large | 100 | 0 | 485 ± 81 | 0.7 ± 8.1 | 107.6 ± 7.9 | 0/6 | 52 (18) | 12.5 ± 1.1 (11)* |
| | DNR-GA3 | Large | 200 | 0 | 343 ± 57 | 19.4 ± 8.1 | 82.3 ± 9.0 | 1/6 | 96 (18) | 57.9 ± 10.9 (9)* |

*$P$ <0.02 when compared with control; $P$ < 0.02 when compared with DNR. [a]n.a., not applicable; [b]tumour volume-doubling time in days from a relative volume of 1 to 4; [c]maximum growth inhibition.

© Cancer Research Campaign 1998

than DNR ($P < 0.01$). Also, the higher dose of 2 × 200 mg kg$^{-1}$ DNR-GA3 was slightly, but not significantly, more effective than the lower dose of 2 × 150 mg kg$^{-1}$ DNR-GA3 (Figure 5). Similar data were obtained for the MRI-H-207 tumour line in which 200 mg kg$^{-1}$ DNR-GA3 on day 0 was more effective than the lower dose of 100 mg kg$^{-1}$ DNR-GA3 on day 0 ($P < 0.002$) (Figure 4 and Table 2).

### DISCUSSION

The objective of this study was to investigate the potential increase in the therapeutic index of the glucuronide prodrug DNR-GA3 when compared with DNR. In vitro, the prodrug was 18-fold less toxic than DNR. In mice bearing human ovarian cancer xenografts, the MTD of DNR-GA3 was six- to tenfold higher than that of DNR. The prodrug was apparently activated in the tumours by β-glucuronidase and inhibited tumour growth in human ovarian cancer xenografts that were sensitive to the parent drug DNR. In these three xenografts (OVCAR-3, A2780, and MRI-H-207), the inhibitory effect at MTD was better than the tumour growth delay obtained with the parent drug.

The different DNR-glucuronide prodrugs synthesized were designed to be rapidly activated in the presence of human β-glucuronidase. DNR-GA3 was most rapidly activated in vitro by human β-glucuronidase (Houba et al, 1996*a*), and was chosen for in vivo analysis. DNR-GA3 is stable in vivo, it is a hydrophilic molecule that hardly passes through the cell membrane into the cell. DNR-GA3 will, therefore, not be activated by intracellular β-glucuronidase. Activation in the circulation is also less likely as the plasma levels of β-glucuronidase are very low (Fishman, 1970). Bosslet et al (1995) and Schumacher et al (1996) have demonstrated high levels of β-glucuronidase in necrotic areas in tumours. Therefore, it could be expected that DNR-GA3 will be activated selectively by human β-glucuronidase released from necrotic tumour cells.

The difference in MTD between DNR-GA3 and DNR in mice bearing human ovarian cancer xenografts may be explained by the more rapid clearance of DNR-GA3 (elimination $t_{1/2}$ = 20 min) than that of DNR (elimination $t_{1/2}$ = 720 min) from the circulation. Thus far, we have no information on the nature of the dose-limiting toxicity in mice. We observed, however, the formation of ascites at higher doses of DNR-GA3 as also described for DNR (Boven et al, 1996). The variation in the MTD of DNR-GA3 found among the four different human ovarian cancer xenografts may possibly be clarified by differences in activation and leakage of DNR from the tumours into the circulation.

The treatment experiments showed that the prodrug DNR-GA3 induced better inhibition of growth in three out of four human ovarian cancer xenografts than equitoxic doses of DNR (OVCAR-3, A2780 and MRI-H-207). This observation may be explained by higher local DNR concentrations in the tumour from activated DNR-GA3. Earlier, it has been demonstrated that there is a steep dose–response curve for anthracyclines (Frei and Canellos, 1980). Bosslet et al (1995) have described that s.c. grown LoVo colon cancer xenografts with a diameter larger than 2 mm had necrotic areas, where β-glucuronidase was present in high concentrations. This group has also demonstrated that a glucuronyl-spacer–DOX prodrug showed better therapeutic effects than DOX.

It was hypothesized that large tumours contain more necrosis and, thus, more β-glucuronidase would be available to activate DNR-GA3. Indeed, we calculated a relatively longer increase in two volume-doubling times for large tumours of the OVCAR-3 and the MRI-H-207 tumour lines when compared with the values of the respective small tumours. With respect to the clinic, this finding is of interest because the treatment of patients with large tumour deposits remains a challenge.

The administration of anthracycline prodrugs to be activated at the tumour site may induce an even better growth inhibition when combined with a second approach: antibody-directed enzyme prodrug therapy (ADEPT; Bagshawe et al, 1988). In ADEPT, prodrugs are activated in the tumour by an administered tumour-specific monoclonal antibody–enzyme conjugate. In our point of view, DNR-GA3 is very suitable for ADEPT. We have shown earlier that a conjugate of monoclonal antibody 323/A3 and human β-glucuronidase bound to tumour cells can activate DNR-GA3 in an efficient manner (Haisma et al, 1992; Houba et al, 1996*b*). If such a tumour-specific conjugate is administered before DNR-GA3 injection, activation could also occur in the non-necrotic smaller tumour lesions.

In conclusion, our findings suggest that the glucuronidated anthracycline DNR-GA3 may have a better therapeutic index in advanced solid tumours in which anthracyclines are considered for treatment.

### ACKNOWLEDGEMENTS

This work was supported by the Dutch Cancer Society. We are grateful to IH van der Meulen–Muileman for technical assistance.

### REFERENCES

Albin N, Massaad L, Toussaint C, Mathieu M-C, Morizet J, Parise O, Gouyette A and Chabot GG (1993) Main drug-metabolizing enzyme systems in human breast tumors and peritumoral tissues. *Cancer Res* **53**: 3541–3546

Bagshawe KD, Springer CJ, Searle F, Antoniw P, Sharma SK, Melton RG and Sherwood RF (1988) A cytotoxic agent can be generated selectively at cancer sites. *Br J Cancer* **58**: 700–703

Bosslet K, Czech J and Hoffman D (1995) A novel one-step tumor-selective prodrug activation system. *Tumor Targeting* **1**: 45–50

Boven E, Winograd B, Fodstad O, Lobbezoo MW and Pinedo HM (1988) Preclinical phase II studies in human tumor lines: a European multicenter study. *Eur J Cancer Clin Oncol* **24**: 567–573

Boven E, Hendriks HR, Erkelens CAM and Pinedo HM (1992) The anti-tumour effects of the prodrugs *N*-L-leucyl-doxorubicin and vinblastine-isoleucinate in human ovarian cancer xenografts. *Br J Cancer* **66**: 1044–1047

Boven E, De Jong J, Kuiper CM, Bast A and Van der Vijgh WJF (1996) Relationship between the tumour tissue pharmacokinetics and antiproliferative effects of anthracyclines and their metabolites. *Eur J Cancer* **32A**: 1382–1387

Chakravarty PK, Carl PL, Weber MJ and Katzenellenbogen JA (1983) Plasmin-activated prodrugs for cancer chemotherapy. 2. Synthesis and biological activity of peptidyl derivatives of doxorubicin. *J Med Chem* **26**: 638–644

Connors TA and Whisson ME (1966) Cure of mice bearing advanced plasma cell tumours with aniline mustard: the relationship between glucuronidase activity and tumour sensitivity. *Nature* **210**: 866–867

De Jong J, Guerand WS, Schoofs WPR, Bast A and Van der Vijgh WJF (1991) Simple and sensitive quantification of anthracyclines in mouse atrial tissue using high-performance liquid chromatography and fluorescence detection. *J Chromatogr* **570**: 209–216

Deprez-de Campeneere D, Baurain R and Trouet A (1982) Accumulation and metabolism of new anthracycline derivatives in the heart after i.v. injection into mice. *Cancer Chem Pharmacol* **8**: 193–197

Fishman WH (1970) *Metabolic Conjugation and Metabolic Hydrolysis*. Academic Press: New York

Frei E and Canellos GP (1980) Dose: a critical factor in cancer chemotherapy. *Am J Med* **69**: 585–590

Haisma HJ, Boven E, Van Muijen M, De Jong J, Van der Vijgh WJF and Pinedo HM (1992) A monoclonal antibody-β-glucuronidase conjugate as activator of the prodrug epirubicin-glucuronide for specific treatment of cancer. *Br J Cancer* **66**: 474–478

© Cancer Research Campaign 1998

Hamilton TC, Young RC, McKoy WM, Grotzinger KR, Green JA, Chu EW, Whang-Peng J, Rogan AM, Green WR and Ozols RF (1983) Characterization of a human ovarian carcinoma cell line (NIH:OVCAR-3) with androgen and estrogen receptors. *Cancer Res* **43**: 5379–5389

Houba PHJ, Leenders RGG, Boven E, Scheeren JW, Pinedo HM and Haisma HJ (1996*a*) Characterization of novel anthracycline prodrugs activated by human β-glucuronidase for use in antibody-directed enzyme prodrug therapy. *Biochem Pharmacol* **52**: 455–463

Houba PHJ, Boven E, Haisma HJ (1996*b*) Improved characteristics of a human β-glucuronidase–antibody conjugate after deglycosylation for use in antibody-directed enzyme prodrug therapy. *Bioconj Chem* **7**: 606–611

Kearney AS (1996) Prodrugs and targeted drug delivery. *Adv Drug Del Rev* **19**: 225–239

Molthoff CFM, Calame JJ, Pinedo HH and Boven E (1991) Human ovarian cancer xenografts in nude mice: characterization and analysis of antigen expression. *Int J Cancer* **47**: 72–79

Schumacher U, Adam E, Zangemeister-Wittke U and Gossrau R (1996) Histochemistry of therapeutically relevant enzymes in human tumours transplanted into severe combined immunodeficient (SCID) mice: nitric oxide synthase-associated diaphorase, β-D-glucuronidase and non-specific alkaline phosphatase. *Acta Histochem* **98**: 381–387

Sinhababu AK and Thakker DR (1996) Prodrugs of anticancer agents. *Adv Drug Del Rev* **19**: 241–273

© Cancer Research Campaign 1998